# A Patella-Sided Tensioning Technique for Medial Patellofemoral Ligament Reconstruction

**DOI:** 10.1016/j.eats.2022.11.017

**Published:** 2023-03-23

**Authors:** Alexander J. Duke, Amos Dai, Daniel Botros, William Leatherwood, Nicholas J. Montemurro, Meghan Richardson, Mark Grossman

**Affiliations:** a Department of Orthopaedic Surgery, Stony Brook University Hospital, Stony Brook, New York, U.S.A.; b Department of Orthopaedic Surgery, NYU-Langone Winthrop Hospital, Mineola, New York, U.S.A.

## Abstract

Reconstruction of the medial patellofemoral ligament (MPFL) is a common procedure for treatment of recurrent patellofemoral instability. Over the last two decades, multiple surgical techniques for MPFL reconstruction have been described with no clear consensus on a superior reconstruction technique. Appropriate graft tensioning is one of the most important factors for a successful MPFL reconstruction. Overtensioning of the MPFL graft can lead to patellofemoral joint overload and undertensioning can lead to recurrent instability. Current literature demonstrates descriptions of MPFL reconstruction with final graft tensioning performed off of the femoral side. We describe a technique in this article for performing final graft tensioning from the patellar side, which gives the surgeon an option for intraoperative tension adjustments after evaluating patellar tracking.

## Technique Video

Video 1 XXX

## Introduction

It is well known that patellar dislocations are one of the most common acute knee disorders.^1^, ^2^, ^3^ Patellofemoral dislocations are defined as the complete loss of contact between the articular surfaces of the patella and femoral trochlea. Dislocations usually occur secondary to a noncontact twisting injury with the knee extended and the foot externally rotated and are less commonly caused by a direct blow.

The medial patellofemoral ligament (MPFL) is the primary stabilizer of the patella between full knee extension and 30° of flexion. Injury to the MPFL is associated with significant morbidity, such as recurrent instability and patellofemoral osteoarthritis, which can ultimately lead to a decline in physical abilities.^3^, ^4^, ^5^, ^6^, ^7^ Following dislocations, recurrent dislocation events have been reported in up to 44% of patients.^8^

While nonoperative treatment had long been considered the standard of care for patellar dislocations, surgical treatment has since become the gold standard recommendation for patients experiencing recurrent patellofemoral instability.^9^, ^10^, ^11^ It is also considered the treatment of choice for those who have failed nonoperative measures of functional rehabilitation.^12^ Two main options are available for surgical intervention of knee instability associated with an MPFL injury: reconstruction or repair. While both improve clinical outcomes compared to nonoperative modalities, reconstruction has been shown to be superior.^13^ Furthermore, since Ellera Gomes reported that MPFL reconstruction is the preferred surgical treatment for recurrent patellar instability, this procedure has been implemented on a large scale both in isolation and in combination with other procedures to correct soft tissue imbalance or bony malalignment.^12^

In the past two decades, multiple surgical techniques for MPFL reconstruction have been described.^6^^,^^12^^,^^14^^,^^15^ They differ both in the type of graft used (patellar tendon vs. quadriceps tendon and autograft vs. cadaver allograft), as well as in the patellar and femoral fixation methods, such as anchors and interference screws, as well as transosseous tunnels.^16^, ^17^, ^18^, ^19^ However, to date, there is no consensus on a superior method of MPFL reconstruction.

Aside from graft choice, proper tunnel position, and varying fixation techniques, appropriate graft tensioning is one of the most important factors for a successful MPFL reconstruction.^20^ Tightening the MPFL graft can overload the patellofemoral joint surface and restrict range of motion, resulting in a postoperative loss of knee flexion—one of the most common complications of this procedure.^21^^,^^22^ In contrast, a “looser” reconstruction can lead to problematic recurrent patellar instability and a subsequent new dislocation at a rate of 0-4%.^6^^,^^14^^,^^22^^,^^23^^,^^24^

At present, several studies have demonstrated successful outcomes after MPFL reconstruction with final graft tensioning on the femoral side.^25^^,^^26^ This article describes an alternative technique of performing final graft tensioning from the patellar side, which provides an option for intraoperative tension adjustments after evaluating patellar tracking.

This study received Institutional Review Board approval (#945327).

## Surgical Technique

**Fig 1:**
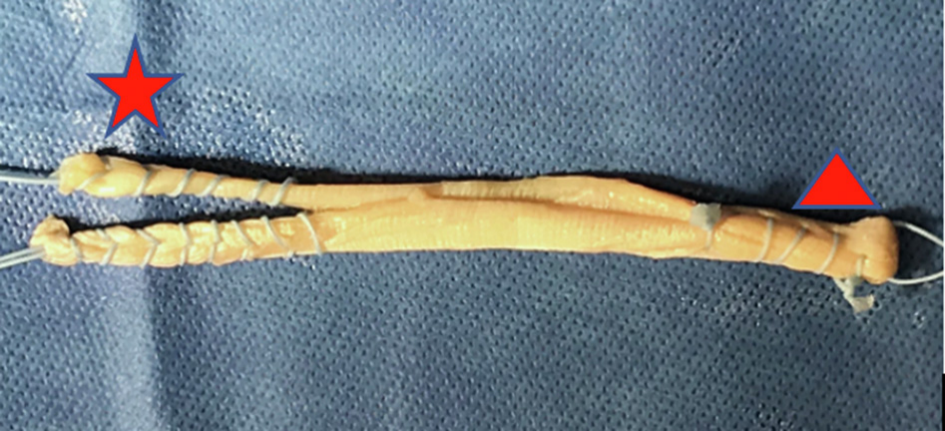
A harvested semitendinosus graft ∼20 cm in length. The tendon has been folded on itself one time and then whipstitched at both the free and folded ends to a length of about 25 mm using a #2 FiberLoop suture (Arthrex, Naples, FL). The femoral side of the graft (triangle) and the patellar side (star) is shown here. The free ends on the patella (star) side are individually whipstitched to allow for adjustable tensioning.

Following a standard diagnostic arthroscopic exam of the knee to ensure no underlying intra-articular occult pathology and to evaluate patellar tracking, a semitendinosus allograft with a desired length of 180-220 mm is prepared. The tendon is folded over on itself one time, and then a whipstitch is placed on each free end of the tendon using a #2 FiberLoop suture (Arthrex, Naples, FL). The doubled-over end is whipstitched together, and 25 mm is measured and marked from the end (Fig 1).

**Fig 2:**
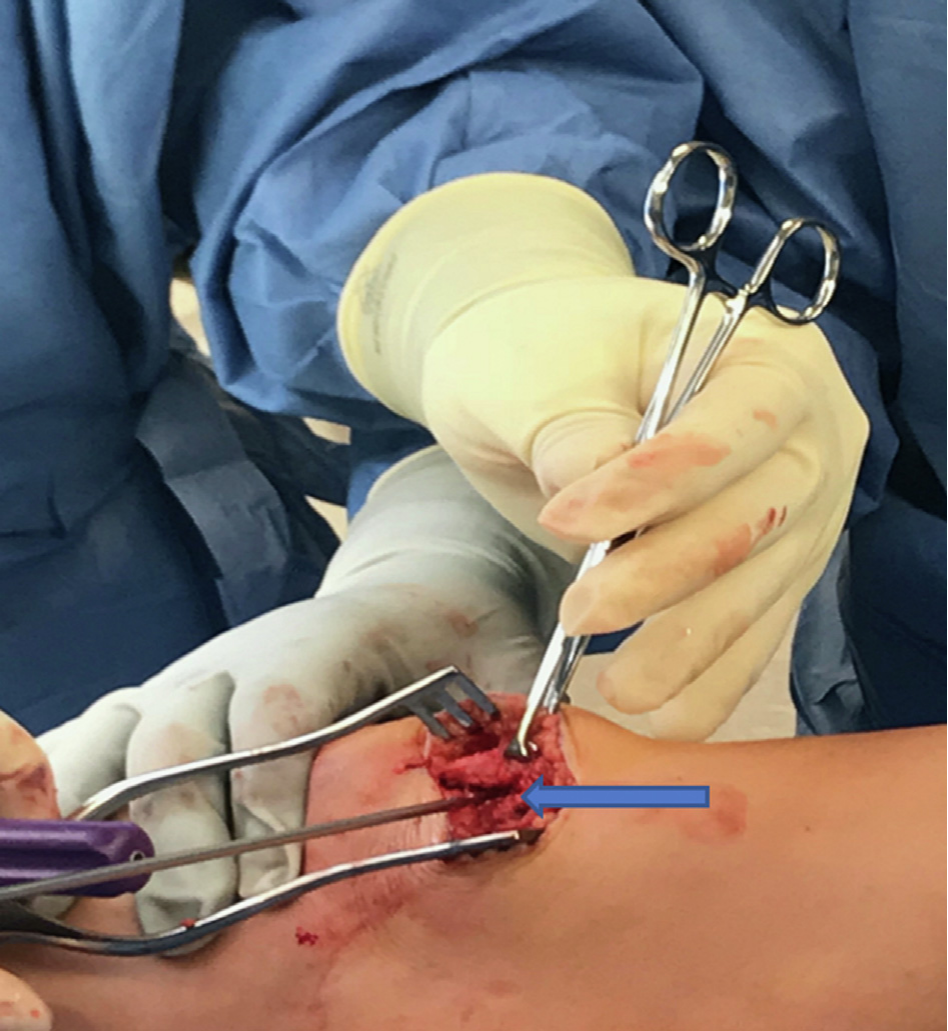
The patient is positioned supine with their head oriented to the right of the image. Exposure of the medial aspect of the patella down to the synovial layer. A trough is then created on the medial aspect of the patella to expose bleeding bone for preparation of BioComposite suture tac placement (arrow). An assistant places counter pressure on the lateral side of the knee, as the patella is drilled and tapped twice for 2 points of fixation.

**Fig 3:**
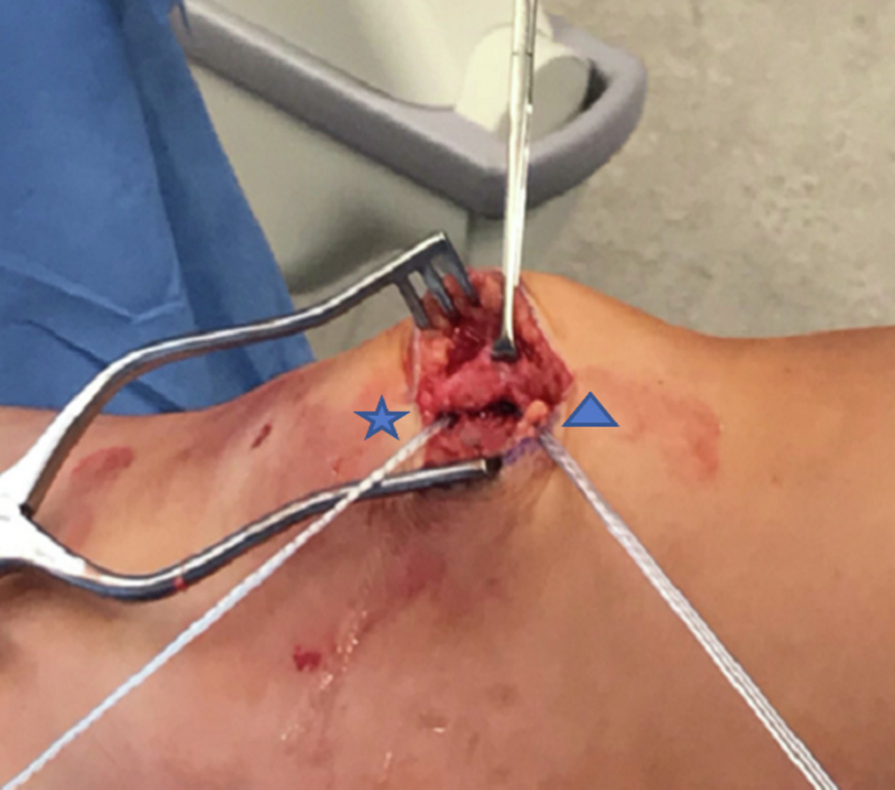
The patient is positioned supine with the patient’s head oriented to the right of the image. The first limb (triangle) of double loaded #2 FiberWire 3.0 mm BioComposite suture tac (Arthrex) is placed in the superomedial aspect of the patella and a second double-loaded #2 FiberWire 3.0 mm BioComposite suture tac (Arthrex) is placed ∼5 mm distal to the first in the patella (star).

A medial incision is made adjacent to the patella, and dissection is carried out until the synovial layer medial to the patella is encountered. A trough is then created on the medial aspect of the patella to expose bleeding bone to receive the graft tissue (Fig 2). One double-loaded #2 FiberWire 3.0-mm biocomposite suture tac (Arthrex) is placed in the superomedial aspect of the patella, and a second double loaded #2 FiberWire 3.0-mm BioComposite suture tac (Arthrex, Naples, FL) is placed ∼5 mm distal to the first in the patella (Fig 3).

**Fig 4:**
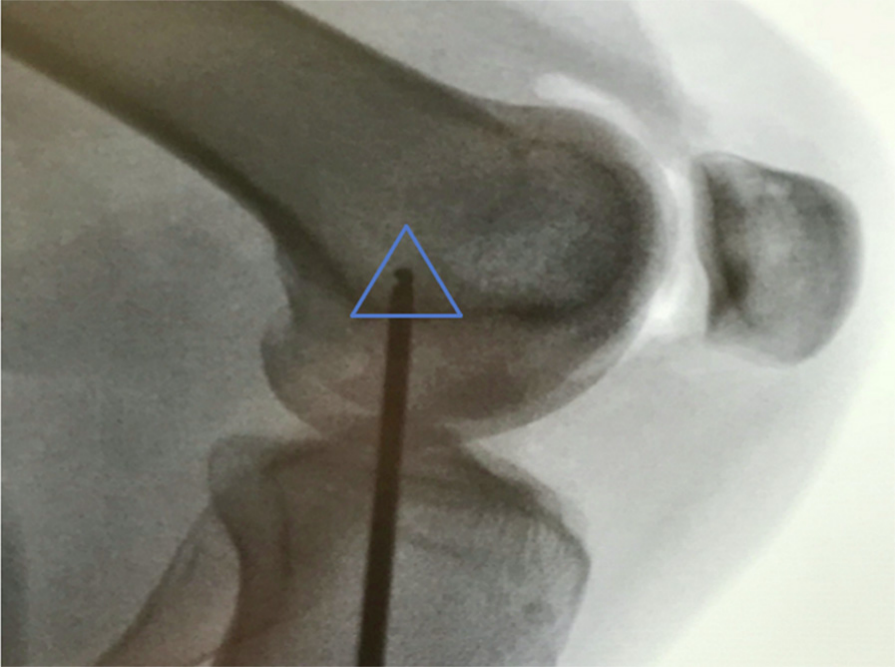
Fluoroscopic image demonstrating Schottle’s point (triangle). This is 1 mm anterior to the posterior cortex extension line, 2.5 mm distal to the posterior origin of the medial femoral condyle, and proximal to the level of the posterior point of the Blumensaat’s line with both posterior condyles projected in the same plane. This represents where the femoral side of the graft will be placed using a 2.4-mm Drill Tip Guide (Beath) pin (Arthrex).

Attention is then turned to the femur. A small incision is made and fluoroscopic imaging is used to locate Schottle’s point (Fig 4). A 2.4-mm Drill Tip Guide (Beath) pin (Arthrex) is placed into the femur from medial to lateral at Schottle’s Point. Schottle’s point is identified, as described by Schottle et al. in 2008, “1 mm anterior to the posterior cortex extension line, 2.5 mm distal to the posterior origin of the medial femoral condyle, and proximal to the level of the posterior point of the Blumensaat line on a lateral radiograph with both posterior condyles projected in the same plane.”^15^ A 7-mm cannulated Headed Reamer (Arthrex) is used to ream 30 mm over the pin in a line-to-line fashion.

**Fig 5:**
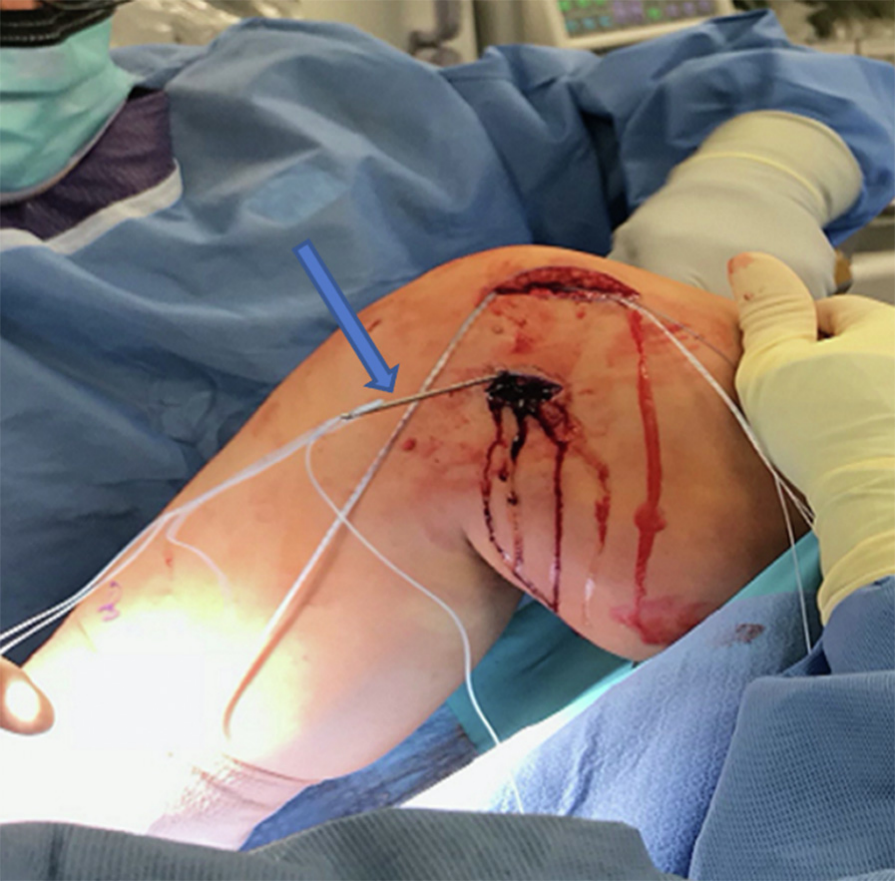
Docking of the lateral distal femur with a 2.4-mm Drill Tip Guide (Beath) pin (Arthrex) (arrow) passing into Schottle’s point. Following this, a 7-mm cannulated headed reamer (Arthrex) is used to ream 30 mm over the pin in a line-to-line fashion (not shown).

**Fig 6:**
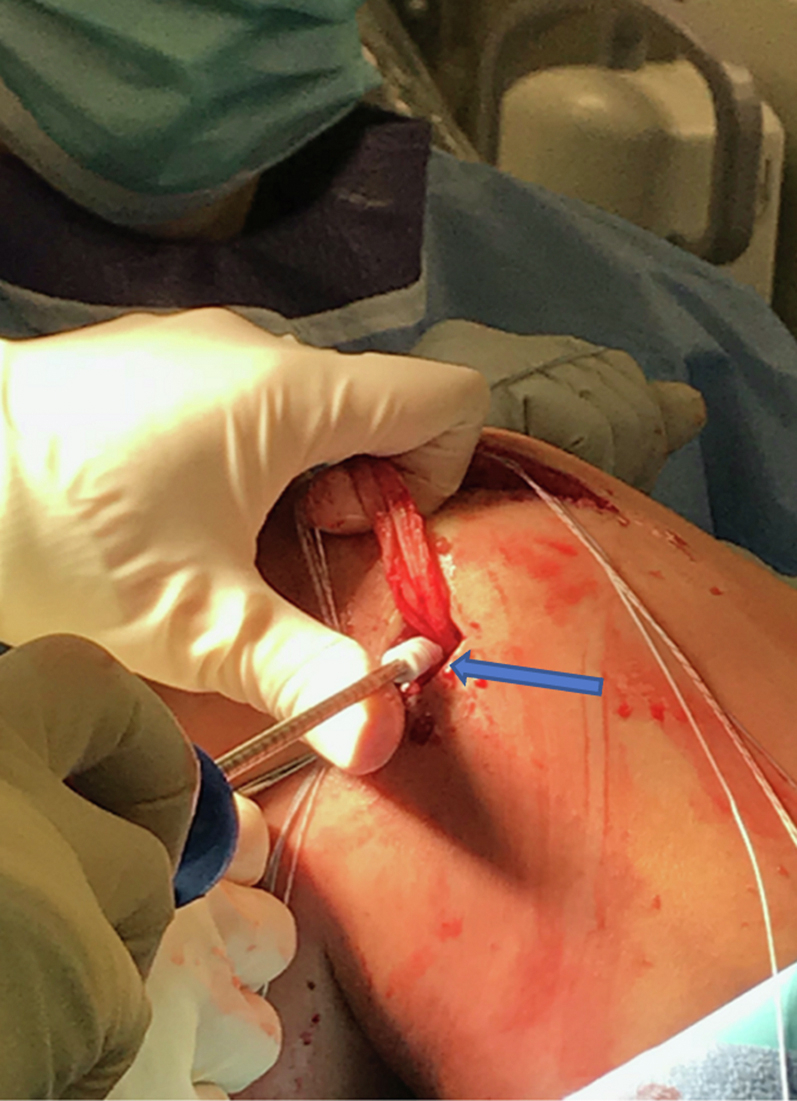
Following insertion of a 2.4-mm Drill Tip Guide (Beath) pin (Arthrex), as well as reaming using a 7-mm cannulated Headed Reamer (Arthrex), the femoral side of the allograft is docked at Schottle’s point using 6 × 23mm FastThread BioComposite interference screw (Arthrex, Naples, FL) (arrow).

The allograft is docked into the femur by passing the sutures of the doubled-over end with the Beath pin through to the lateral side of the femur (Fig 5). Care is taken to ensure that the graft is fully docked by confirming that the marked 25-mm line is within the reamed canal. A 1.1 nitinol GuideWire is placed into the canal and a 6 × 23 mm FastThread BioComposite interference screw (Arthrex) is placed to secure the femoral portion of the graft (Fig 6).

**Fig 7:**
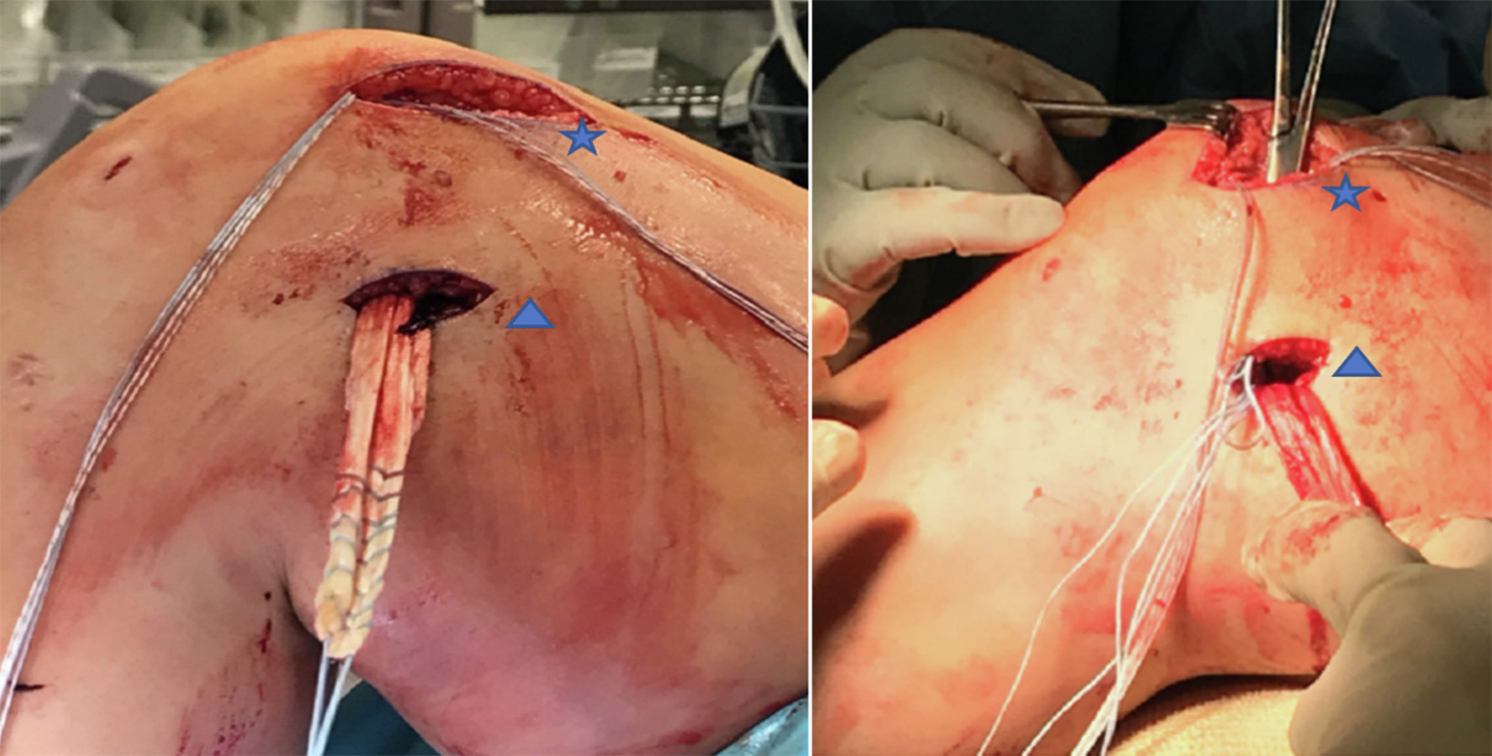
The semitendinosus graft is now shuttled underneath the skin and subcutaneous tissue from the femoral incision (triangle) to the patellar (star). A bump has been placed under the knee to achieve 30° of flexion for this part of the technique.

**Fig 8:**
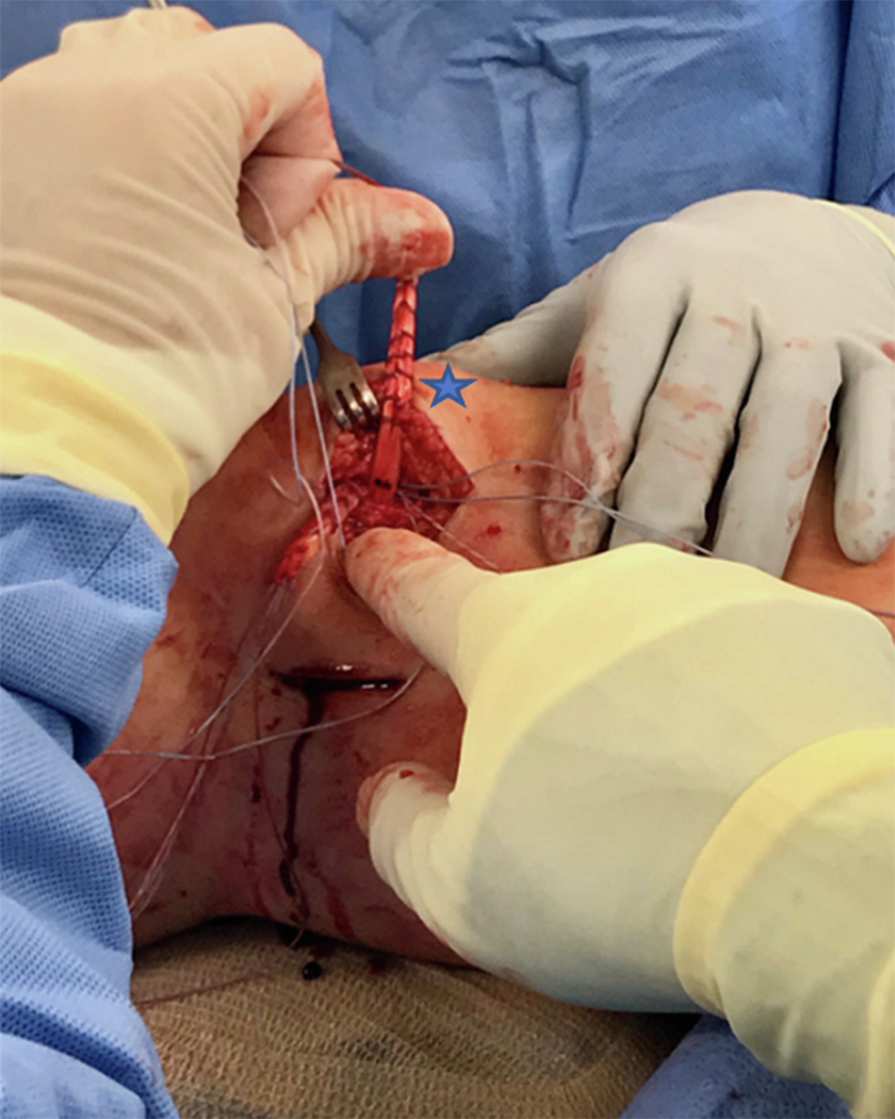
Tensioning of superomedial (star) limb as assistant places pressure to the lateral patella. The patella is held steady manually within the trochlea, and tension is set by suturing the first limb of the graft into place (proximal first) using the double-loaded suture tac ends and a free needle. The first limb acts as a fixation point and is sutured in place with care not to overtension. This allows the second limb to optimize the final tension after the knee is taken through a range of motion with critical evaluation of patellar tracking. Of note, the two suture limbs are placed tensioned in different planes as to avoid cutout. Once the graft is secured, the free ends are trimmed (not shown).

A bump is placed under the knee to achieve 30° of flexion, and the two free ends of the graft are passed to the medial patella incision (Fig 7). The patella is held steady manually within the trochlea, and tension is set by suturing the first limb of the graft into place (proximal first) using the double-loaded suture tac ends and a free needle (Fig 8). The first limb acts as a fixation point and is sutured in place. It is important to not overtension the first limb, as this would defeat the purpose of the second limb. The purpose of the first limb is to grossly fixate the patella in a range of normal to slight undertension. This allows the second limb to optimize the final tension after the knee is taken through a range of motion with critical evaluation of patellar tracking. When suturing the graft, it is important to place the two limbs of suture in two different planes, so as to avoid the sutures from cutting out longitudinally. Once the graft is secured, the free ends are trimmed (Video 1). Lastly a 0 Vicryl (Ethicon, Somerville, NJ) stitch is used to suture the remnants of the native MPFL over the graft.

The incisions are closed with 2-0 Vicryl (Ethicon), 4-0 Monocryl (Ethicon), and Dermabond (Ethicon). The patient may weight-bear as tolerated with a Bledsoe brace locked in extension immediately.

### Postoperative Rehabilitation

Postoperative rehabilitation consisted of a knee immobilizer locked in extension for 2 weeks and weight bearing as tolerated using crutches for 6 weeks. Range of motion exercises and isometric quadriceps strengthening (quadriceps setting and straight leg raising) were performed immediately and without restriction. Full weight bearing, full knee range of motion, and progressive strength and proprioceptive training were allowed after 6 weeks. Range of motion exercises were also initiated from the second day after surgery and gradually progressed. Goals for knee flexion are 90° by the fourth week and 120° was by the sixth week. Functional activities, including walking, jogging and running, were introduced at 3 months. Sports activities were initiated after 3 to 4 months of surgery and according to muscular and functional recovery. Unrestricted participation was allowed after 6 months, provided that the patient has a pain-free knee, full range of motion, normal patellar stability, and normal or near-normal quadriceps strength.

## Discussion

The MPFL is an important soft-tissue restraint of the patellofemoral joint, providing up to 60% of stability to resisting lateral patellar displacement at 0° to 30° of knee flexion.^4^ The MPFL is ruptured in as many as 90% of acute patella dislocations and is either ruptured or attenuated in almost 100% of cases of recurrent instability.^15^ Earlier literature reported an uncomplicated natural history of traumatic patellar dislocation, but newer studies showed that 30–50% of patients will continue to suffer redislocations or anterior knee pain, and more than 50% will experience limitations during sports activities.^5^^,^^27^^,^^28^ Restoration of MPFL integrity, including its anatomic insertion sites, can restore patellofemoral tracking to normal after injury and is an important component of any surgical plan for patellar stabilization.^29^^,^^30^

A recent review reported an overall cumulative complication rate of 26.1%, including restriction of range of motion, medial instability by overtightening, fracture, and recurrent lateral instability.^31^^,^^32^ Most complications are due to two critical surgical technical points: the femoral tunnel position and graft tension. Graft tunnel malposition may change the mechanical tension and tracking pattern and affect clinical outcome. Anatomical placement of the femoral tunnel can be reached by identifying the adductor tubercle and femoral epicondyle and can be furthered confirmed by using Schottle’s point on fluoroscopy. With the advanced anatomical understanding gained by Schottle’s point, graft tension has become one of the more important concerns for successful MPFL reconstruction. Recurrent instability can occur when the graft is too loose or if fixation at either the femoral or patellar side fails. Conversely, overtightening or malpositioning of the graft can lead to maltracking, stiffness, and excessive medial compressive forces. Appropriate tensioning of graft is essential to maintain stability at different flexion angles.^33^ Malpositioning the graft and underestimating graft length may as much as double the graft tension in flexion, which is likely to lead to the development of stiffness and patellofemoral arthrosis.^34^^,^^35^

Our described technique for MPFL reconstruction is designed to decrease the risk of complications associated with the aforementioned technical errors. This technique incorporates several aspects of previously described successful techniques, including careful identification of the anatomic femoral insertion site using verified radiographic landmarks. We perform final graft tension on the patellar side of the knee, while directly (with manual testing) checking the stability and patellofemoral alignment. We believe that this technique allows more control to the surgeon and excellent results by allowing for adjustment of tension through insertion of the second patellar limb of the graft after cycling the knee (Table 1).

**Table 1:** Advantages and Disadvantageous of Patellar Sided Tensioning for MPFL Reconstruction

Advantages	Disadvantages
• Allows for graft tensioning and cycling of the knee after Schottle’s point has been established • Allows surgeon to fine tune tensioning by using second limb of the patella • Allows for graft shortening following tensioning if excess graft harvested • Allows for different amount of tensioning between the two patellar limbs to address the varying amount of instability throughout range of flexion of the knee	• Lack of biomechanical studies to suggest superiority of patellar tensioning over femoral tensioning • Unequal tensioning of the two patellar limbs may lead to medial-lateral motion of the patella during knee cycling • Asymmetric tensioning of the patellar limbs may lead to construct failure

Future studies should attempt a comparative clinical study of this MPFL reconstruction tensioning technique to a femoral sided tensioning technique and a conservative physical therapy approach to determine if there is a clinical superiority of this described method. With safety and efficacy of tensioning off the patellar side established, biomechanical cadaver studies should be conducted to demonstrate whether this described method is more superior than tensioning off of the femoral side. Several pearls and pitfalls for this technique can found in Table 2. Another area that future studies should further evaluate is the allograft choice. Recently, quadriceps tendon has been demonstrated to be an alternative graft choice with good results.^36^ However, similar to semitendinosus allograft, success still depends on appropriate tensioning of graft. Ultimately, the main aim of this study was to highlight a technique for anatomic MPFL reconstruction and present its clinical results.

**Table 2:** Pearls and Pitfall

Pearls	Pitfalls
• Slightly under tension the first patellar side attachment to allow for final tightening with the second • Attach superior patellar site first to give more uniform tracking	• Over tensioning the first patellar attachment, which would not allow final adjustment with the second • Incidentally making the patellar sided arms of graft different lengths, which may not allow you secure graft and adjust tension with both limbs

## Supplementary Data

Video 1 Intraoperative video demonstrating step-by-step technique for tensioning during medial patellofemoral ligament (MPFL) reconstruction. A standard diagnostic arthroscopic exam of the knee is performed. A semitendinosus allograft is prepared on the back table. The prepared graft has a doubled-over end on one side and two free limbs on the other side. A medial parapatellar dissection is performed down to the synovial layer and a trough is created on the medial aspect of the patella. Two double-loaded suture anchors are placed 5 mm apart on the superomedial edge of the patella. The doubled-over end of the graft is docked into the femur at Schottle’s point. This is located using intraoperative fluoroscopy. A 6 × 23 mm BioComposite interference screw to secure the femoral portion of the graft. Next, the free ends of the graft are tunneled under the skin bridge toward the medial parapatellar incision. The knee is flexed to 30° using a bump. The first suture limb is pulled up against the patella to the desired tension. The graft is then marked where it meets the proximal suture anchor. Sutures from the first suture anchor are then passed through the free graft limb using a free needle. The solid blue sutures are tied first. Then the striped blue sutures. Excess graft is sharply excised. The patella is taken through a range of motion to assess stability. The final graft tension is now set with the second limb in a similar fashion.

ICMJE author disclosure forms
